# MRCP compared to diagnostic ERCP for diagnosis when biliary obstruction is suspected: a systematic review

**DOI:** 10.1186/1471-2342-6-9

**Published:** 2006-08-14

**Authors:** Eva C Kaltenthaler, Stephen J Walters, Jim Chilcott, Anthony Blakeborough, Yolanda Bravo Vergel, Steven Thomas

**Affiliations:** 1 School of Health and Related Research, University of Sheffield, Regent Court, 30 Regent Street, Sheffield S1 4DA, UK; 2 Royal Hallamshire Hospital, Glossop Road, Sheffield S10 2JF, UK; 3 Centre for Health Economics, University of York, York YO10 5DD, UK; 4 Northern General Hospital, Herries Road, Sheffield S5 7AU, UK

## Abstract

**Background:**

Magnetic resonance cholangiopancreatography (MRCP) is an alternative to diagnostic endoscopic retrograde cholangiopancreatography (ERCP) for investigating biliary obstruction. The use of MRCP, a non-invasive procedure, may prevent the use of unnecessary invasive procedures. The aim of the study was to compare the findings of MRCP with those of ERCP by the computation of accuracy statistics.

**Methods:**

Thirteen electronic bibliographic databases, covering biomedical, science, health economics and grey literature were searched. A systematic review of studies comparing MRCP to diagnostic ERCP in patients with suspected biliary obstruction was conducted. Sensitivity, specificity, likelihood ratios, acceptability and adverse events were reported.

**Results:**

25 studies were identified reporting several conditions including choledocholithiasis (18 studies), malignancy (four studies), obstruction (three studies), stricture (two studies) and dilatation (five studies). Three of the 18 studies reporting choledocholithiasis were excluded from the analysis due to lack of data, or differences in study design. The sensitivity for the 15 studies of choledocholithiasis ranged from 0.50 to 1.00 while specificity ranged from 0.83 to 1.00. The positive likelihood ratio ranged: from 5.44–47.72 and the negative likelihood ratio for the 15 studies ranged from 0.00–0.51. Significant heterogeneity was found across the 15 studies so the sensitivities and specificities were summarised by a Receiver Operating Characteristic (ROC) curve. For malignancy, sensitivity ranged from 0.81 to 0.94 and specificity from 0.92 to 1.00. Positive likelihood ratios ranged from 10.12 to 43 and negative likelihood ratios ranged from 0.15 to 0.21, although these estimates were less reliable.

**Conclusion:**

MRCP is a comparable diagnostic investigation in comparison to ERCP for diagnosing biliary obstruction.

## Background

Biliary obstruction may be due to a variety of causes including choledocholithiasis, tumours, and trauma, including injury after gall bladder surgery, with choledocolithiasis being the most common cause. The prevalence of gallstones in England and Wales was 182 per 10,000 person years at risk. The incidence rate was 8 per 10,000 person years at risk for 1991–1992 [1]. Patients with suspected biliary obstruction present with abnormal liver function and symptoms such as jaundice, pale-coloured stools, dark urine, itching, abdominal pain in the upper right quadrant, fever, nausea and vomiting. Endoscopic ultrasonography (EUS) is the first-line imaging investigation in patients with jaundice or right upper-quadrant pain [2]. Although EUS is non-invasive, quick and inexpensive it is very operator and patient dependent.

Endoscopic retrograde cholangiopancreatography (ERCP) is currently the 'gold standard' for the diagnosis of biliary obstruction. It is one of several invasive direct cholangiography techniques. However, it is an imperfect diagnostic tool and other procedures may be more appropriate gold standards for diagnosis in the future [3]. Magnetic resonance cholangiopancreatography (MRCP) is an alternative to diagnostic ERCP for imaging the biliary tree and investigating biliary obstruction. MRCP was developed in 1991 and techniques are continuing to improve. A major feature of MRCP is that it is not a therapeutic procedure, while in contrast ERCP is used for both diagnosis and treatment. MRCP also does not have the small but definite morbidity and mortality associated with ERCP. The use of MRCP in diagnosing biliary obstruction may avoid the use of unnecessary invasive procedures such as ERCP.

Indications for the use of MRCP include: unsuccessful or contraindicated ERCP; patient preference for non-invasive imaging; patients considered to be at low risk of having pancreatic or biliary disease; patients where the need for therapeutic ERCP is considered unlikely; and those with a suspected neoplastic cause for pancreatic or biliary obstruction [4]. No patient preparation is required for MRCP and sedation is not usually required. MRCP is particularly useful where ERCP is difficult, hazardous or impossible. It is also an important option for patients with failed ERCPs. ERCP and MRCP have different contraindications allowing them to be used as complementary techniques.

In order to determine the sensitivity and specificity of MRCP compared to ERCP, a systematic review was undertaken to identify all relevant studies comparing the two techniques using clearly defined inclusion and exclusion criteria. This paper therefore compares the findings of MRCP with diagnostic ERCP for the investigation of biliary obstruction, using accuracy statistics. We also report study quality, population characteristics and suspected conditions. The paper summarises the key clinical points reported in a recent Health Technology Assessment Monograph [5]. Since the most common cause of biliary obstruction is choledocholithiasis, we have concentrated mainly on the diagnosis of this condition.

## Methods

We searched 13 electronic databases Medline, Embase and the Cochrane Controlled Trials Register from inception to January 2003. Reference lists of relevant articles were hand searched and various health services research-related resources were consulted via the Internet. Search terms included population search terms such as biliary, biliary tract, bile, gallbladder, choledocholithiasis and were combined with intervention terms such as magnetic resonance imaging, MRI and non-invasive diagnostic imaging. The search strategy is described in detail elsewhere [5]. No language or study/publication-type restrictions were applied to the searches. Inclusion criteria were adult patients with suspected biliary obstruction or dilatation, as defined by the individual studies, having MRCP and ERCP for diagnostic purposes. Outcome measures included sensitivity, specificity and likelihood ratios in different patient groups, acceptability to patients and adverse effects. The ERCP test results were assumed to be a true 'gold standard' diagnosis, although even the results of this test may be subject to error and thus not represent the patient's true condition. Only English language papers were selected. Studies involving pancreatic ductal system abnormalities were excluded, as were those not including a comparison of MRCP with diagnostic ERCP. Other exclusion criteria were: papers published before 1995; comparison of MRCP with failed or unsuccessful ERCP; studies where MRCP results informed decision to proceed to ERCP; and retrospective study design. Excluded studies were documented together with reasons for exclusion [5]. Data was extracted by one researcher using a standardised data extraction form and checked by another. Full details of the review process are described elsewhere [5].

The studies were assessed using quality criteria for diagnostic or screening tests [6]. These criteria include 14 components of study quality such as: appropriate spectrum of patients, selection criteria, independent assessment of test results, verification bias (whether all patients had both tests), reporting of uninterpretable results and withdrawals among others.

Standard tests for heterogeneity were conducted [7]. Point estimates and 95% confidence intervals for summary statistics (sensitivity, specificity, likelihood ratio) were calculated for each study and presented graphically with forest plots. In meta-analyses of diagnostic results one must also consider variation introduced by changes in the diagnostic threshold; studies may use different thresholds to define positive and negative test results. In the event of heterogeneity and/or variation in the diagnostic threshold, studies were summarised graphically with a Summary Receiver Operating Characteristic curve (SROC) curve and via the Littenberg and Moses (L-M) method for estimation of a best fitting SROC curve [8]. The L-M method consists of linear regression of the log diagnostic odds ratio, *D*, against the log of the measure of diagnostic threshold *S*, to produce estimates of the parameters a and b from the regression equation, *D *= a + b*S*. D and S are calculated from the true positive and false positive rates. We computed the sensitivity and specificity values from the SROC at the mean, minimum and maximum values of the S parameter, to indicate a central value and associated band with which the data were compatible.

## Results

**Figure 1:**
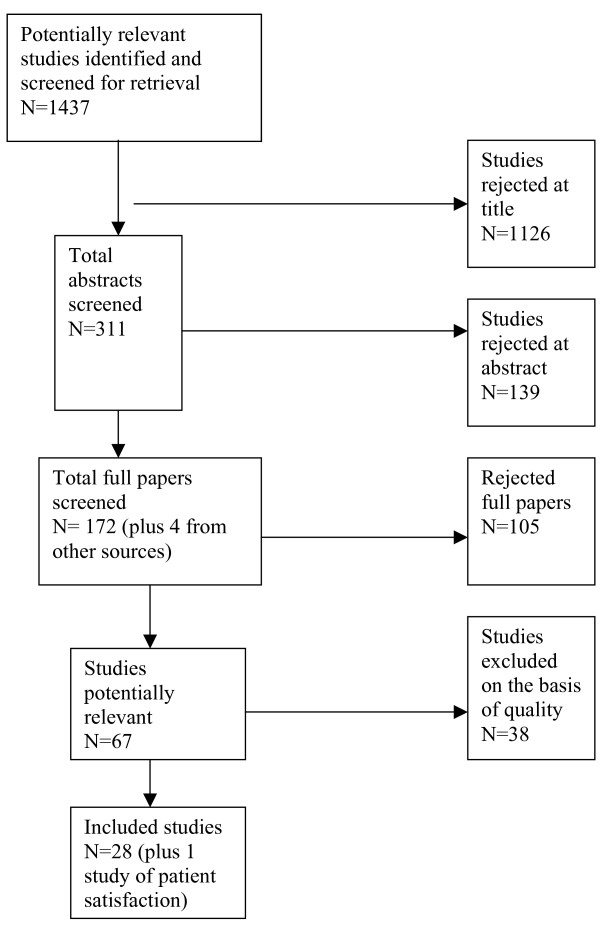
Summary of study selection.

Out of a total of 1437 potentially relevant studies, 28 studies were identified that directly compared MRCP with diagnostic ERCP [9-36]. An additional study was identified that covered patient satisfaction [37]. Study selection is outlined in Figure 1. The quality of studies was variable. Results are shown in Table 1.

In only one study did all selected patients have both MRCP and diagnostic ERCP [31], indicating potential verification bias. Thirteen studies [10-12,14,15,17,18,25,28-30,34,36] reported adequate blinding and only six [10,18,22,27,29,34] reported information on agreement of MRCP results for more than one investigator. Nine studies [10,12,17,20,22,25,28,29,32] gave no information on other diagnostic tests and most studies did not adequately report inclusion and exclusion criteria.

Seven studies [9,17,21,22,25,26,34], reported results comparing MRCP to final diagnosis (including ERCP and other test results) but only four of these reported data comparing MRCP with final diagnosis and ERCP with final diagnosis. The remaining 21 reported results comparing MRCP with diagnostic ERCP. Three [10,17,19] of the 28 studies did not provide enough information to calculate sensitivity, specificity and likelihood ratios. Accuracy was assessed separately for each condition (choledocolithiasis, malignancy, dilatation, obstruction and stricture). The results of the remaining 25 studies are shown in Table 2. Table 3 describes study characteristics. Studies comparing MRCP and ERCP with final diagnosis are shown in Table 4.

### Assessment of effectiveness by condition: choledocolithiasis

**Figure 2:**
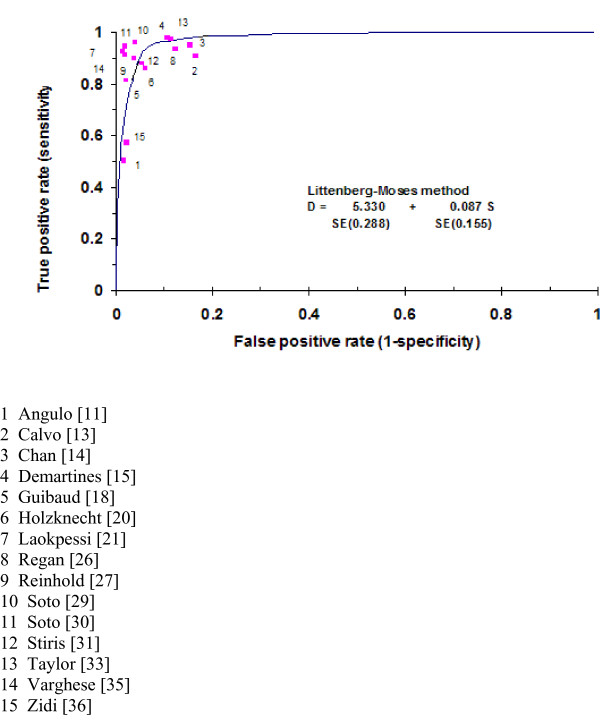
Scatterplot of sensitivity vs. 1-specificity of MRCP test for diagnosing choledocholithiasis with Littenberg-Moses Summary ROC curve and actual data (n = 15 studies).

One of the most common causes of biliary obstruction is choledocolithiasis: indeed 18 out of the 28 studies (64%) were for this condition. For this reason we concentrate mainly on the analysis of these studies. Of the 18 studies reporting results for choledocolithiasis, 15 [11,13-15,18,20,21,26,27,29-31,33,35,36] reported adequate data for analysis. Figure 2 shows a scatterplot of sensitivity vs. specificity for the 15 studies reporting choledocholithiasis.

**Figure 3:**
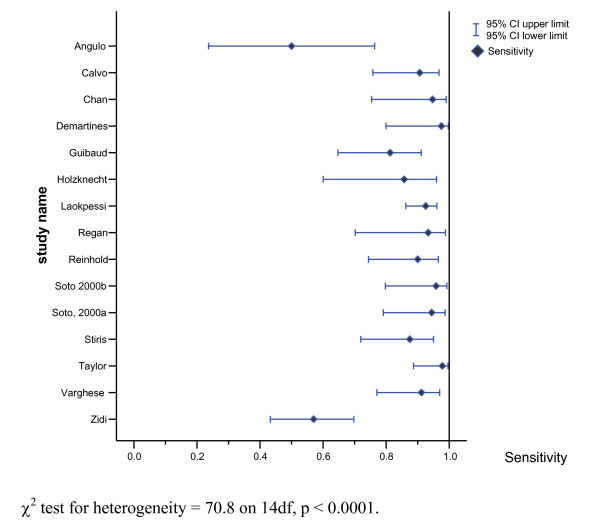
Forest plot of estimated sensitivities of MRCP test for diagnosing choledocholithiasis (n = 15 studies).

**Figure 4:**
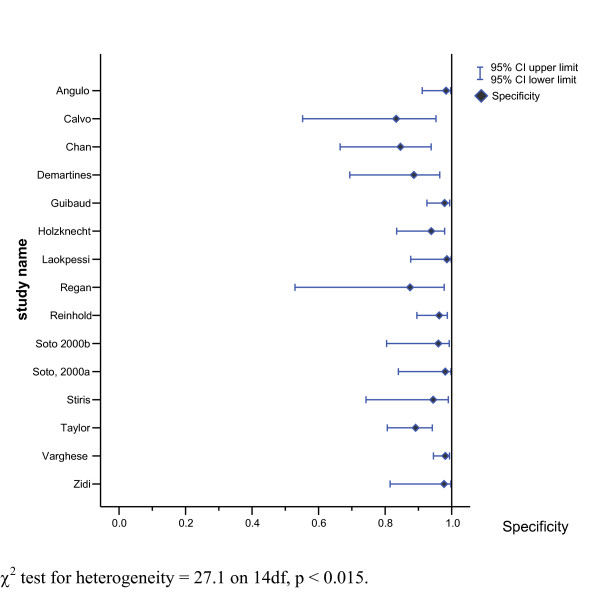
Forest plot of estimated specificities of MRCP test for diagnosing choledocholithiasis (n = 15 studies).

Two of these studies stand out as having sensitivities somewhat lower than the other 13 studies [11,36]. Sensitivities and specificities along with 95% CI for these estimates for the 15 choledocholithiasis studies are presented in Figures 3 and 4.

The sensitivity for the 15 studies of choledocholithiasis ranged from 0.50 to 1.00 while specificity ranged from 0.83 to 1.00. The positive likelihood ratio ranged from 5.44–47.72 and the negative likelihood ratio for the 15 studies ranged from 0.00–0.51. All of the confidence intervals overlap, although the confidence intervals for some studies are wide. Again two studies [11,36] have point estimates in Figure 2 that are clearly different from the other 13 studies, suggesting that theses two studies are outliers. There is also some evidence of statistically significant heterogeneity between studies, (Figure 3 and 4), which suggested that the computation of a SROC curve was the most appropriate way to pool the results of studies.

### Summary ROC curves for diagnosis of choledocholithiasis

Figure 2 also shows the parameter estimates and the results of the Littenberg-Moses method for the estimation of a summary best fitting ROC curve. This curve shows the relationship between sensitivities and specificities across the 15 studies. The non-significant result for the S coefficient estimate of 0.057 [CI: -0.25 to 0.42; p = 0.506], suggests that there is no reliable statistical evidence that the diagnostic odds ratio changes with threshold.

There is no unique joint summary estimate of sensitivity and specificity suitable for use in clinical practice from this plot. Table 5 shows values of sensitivity and specificity off the fitted ROC curve to demonstrate the range of values that the data are compatible with.

### Assessment of effectiveness by other conditions: malignancy, dilatation, obstruction and stricture

For malignancy (three studies [9,18,22]), sensitivity ranged from 0.81 to 0.94 and specificity from 0.92 to 1.00. Positive likelihood ratios ranged from 10.12 to 43 and negative likelihood ratios ranged from 0.15 to 0.21. Although, from the results presented in Table 2 it is apparent that the results for malignancy are much less reliable than those for the other conditions presented. The sensitivity for dilatation (five studies [11,12,20,25,28]) ranged from 0.87 to 1.00 and the specificity from 0.91 to 1.00. For obstruction (three studies [11,18,27]), sensitivity ranged from 0.91 to 1.00 and specificity from 0.91 to 1.00. Sensitivity for stricture (two studies [24,33]) was 1.00 and specificity ranged from 0.98 to 0.99.

### Adverse events and satisfaction with procedures

None of the 28 studies reported any adverse events associated with MRCP. Six studies [9,11,13,27,31,34] reported adverse effects associated with ERCP, including pancreatitis, bleeding and pain. Two [10,15] reported that no adverse events had occurred and 20 gave no information at all regarding adverse events. Claustrophobia associated with MRCP was reported in ten studies [9,11,16,18,19,24,26,27,30,33].

The separate study [37] dealing with patient satisfaction found that most patients preferred MRCP, although there were still some patients who preferred ERCP. Almost half of the patients in this small study complained of claustrophobia associated with MRCP, although very few (5.9%) refused MRCP for this reason.

## Discussion

This systematic review shows that there is evidence that MRCP stands up well to comparisons with diagnostic ERCP, for the diagnosis of many biliary abnormalities. From the small number of comparative studies with final diagnosis, it appears that ERCP is an adequate reference standard for choledocholithiasis with sensitivities and specificities above 89%, however the results for malignancy were much less reliable. The limited evidence on patient satisfaction shows that patients prefer MRCP to diagnostic ERCP. The results of our review are similar to those found by Romagnuolo et al [38] who in their meta-analysis showed high levels of sensitivity and specificity for demonstrating the level and presence of biliary obstruction.

The main advantage of MRCP is that diagnostic ERCP may be associated with significant morbidity and mortality [39]. Reported complication rates of diagnostic ERCP are 5–6% and mortality figures range from 0.01% (36) to 0.89% [40]. Therapeutic ERCP has a complication rate of 4–10% [41]. Diagnostic ERCP has the potential to allow a therapeutic procedure to be performed immediately, but its indiscriminate use will result in an increasing proportion of patients in whom such intervention is found to be unnecessary. If preliminary tests, such as EUS or computed tomography, clearly indicate the need for therapeutic ERCP, then the use of diagnostic MRCP is probably unwarranted. Those patients with a high probability of choledocholethiasis on the basis of EUS investigations usually proceed directly to ERCP. These issues are elaborated in Bravo et al [42].

There were no reported adverse events associated with MRCP, other than claustrophobia. However, in certain circumstances MRCP cannot be performed due to contraindications to Magnetic Resonance Imaging, e.g. in patients with cardiac pacemakers or cochlear implants. Severe claustrophobia may make patients intolerant of the procedure.

### Limitations of this study

Overall the quality of the studies was variable. In only one study did all selected patients have both MRCP and diagnostic ERCP. The reasons why all patients in the other studies did not receive both investigations were not clear. In 21 of the studies, the stated comparison was with ERCP, while in the other seven studies, comparison was with final diagnosis; making comparisons between all studies difficult.

We can consider three ways of categorising a patient: their true condition, the diagnosis and the test results [43]. We have calculated the sensitivity and specificity of MRCP in relationship to diagnosis by ERCP, but we do not necessarily know that the diagnosis is always correct. ERCP is not a perfect gold standard, so differences in diagnosis between MRCP and ERCP may not be due to MRCP giving an incorrect result, but rather to ERCP giving an incorrect result. So we have evaluated MRCP's ability to predict the diagnosis of choledocholithiasis rather than the patient's true disease status. So any errors in the ERCP reference test may lead to either underestimates or overestimates of MRCP's accuracy.

There are several problems associated with using summary ROC curves. For example, although the production of a summary ROC curve does allow the computation of a summary estimate of diagnostic performance, the results cannot be directly applied to clinical practice.

Our results indicate that MRCP is accurate for diagnosis of biliary abnormalities compared to diagnostic ERCP, within the limits of the available data. Good quality studies, particularly randomised controlled trials (we found no comparative clinical trials of the two techniques in our review), are needed comparing MRCP with diagnostic ERCP to final diagnosis, stating inclusion/exclusion criteria and relevant patient characteristics. These studies need to include the full range of target conditions, in particular the differentiation of benign and malignant strictures and the impact on management and outcome. Studies are also needed comparing MRCP with final diagnosis where ERCP is unsuitable or impossible. More research is also needed in the area of patient satisfaction and ways to reduce problems with claustrophobia.

## Conclusion

MRCP is a comparable diagnostic investigation in comparison to ERCP for diagnosing biliary abnormalities. Results were particularly favourable for choledocholethiasis and less so for malignancy. Limited information on patient satisfaction found that patients prefer MRCP to ERCP. The use of MRCP in suitable patients reduces the need for diagnostic ERCP which is associated with significant morbidity and mortality.

## Abbreviations

DOR diagnostic odds ratio

ERCP endoscopic retrograde cholangiopancreatography

L-M Littenberg and Moses

MRCP magnetic resonance cholangiopancreatography

ROC Receiver Operating Characteristic curve

SROC Summary Receiver Operating Characteristic curve

## Competing interests

The author(s) declare that they have no competing interests.

## Authors' contributions

EK carried out the systematic review and prepared the manuscript. SJW carried out the statistical analysis and contributed to the manuscript writing. JC and YBV helped to draft the manuscript. ST and AB provided clinical advice and commented on the manuscript. All authors read and approved the final manuscript.

## Pre-publication history

The pre-publication history for this paper can be accessed here:



## Figures and Tables

**Table 1:** QUADAS quality assessment checklist applied to MRCP studies

**Study**	**1. Patient spectrum**	**2. Selection criteria**	**3. Reference standard**	**4. Time period**	**5. Verification bias**	**6. Same RS**	**7. RS independent of IT**	**8. IT described in detail**	**9. RS described in detail**	**10. IT interpreted without RS**	**11. RS interpreted without IT**	**12. All clinical data**	**13. All test results**	**14. Withdrawals**
Adamek[9]	yes	yes	yes	unclear	no	yes	N/a	yes	no	yes	yes	yes	Yes	yes
Angulo[11]	yes	yes	unclear	yes	no	yes	N/a	yes	no	yes	yes	no	Yes	yes
Barish[12]	yes	yes	unclear	yes	no	yes	N/a	yes	no	yes	unclear	no	yes	yes
Calvo[13]	unclear	yes	unclear	no	no	yes	N/a	yes	unclear	yes	unclear	yes	Yes	yes
Chan[14]	yes	unclear	unclear	yes	no	yes	N/a	yes	unclear	yes	yes	no	Yes	yes
Demartines [15]	yes	yes	unclear	unclear	no	yes	N/a	yes	yes	yes	yes	no	no	no
Dwerryhouse[16]	unclear	yes	unclear	no	no	yes	N/a	yes	no	unclear	yes	unclear	Yes	yes
Guibaud [18]	yes	yes	unclear	no	no	yes	N/a	yes	no	yes	yes	no	Yes	yes
Holzknecht[20]	unclear	yes	unclear	no	no	yes	N/a	yes	yes	yes	yes	unclear	Yes	yes
Laokpessi [21]	no	yes	yes	yes	no	yes	N/a	yes	no	yes	unclear	unclear	Yes	yes
Lee[22]	yes	yes	yes	no	no	yes	N/a	yes	yes	yes	yes	unclear	No	yes
Lomanto [23]	yes	unclear	unclear	unclear	no	yes	N/a	yes	no	unclear	unclear	unclear	No	no
Lomas[24]	yes	yes	unclear	yes	no	yes	N/a	yes	yes	no	no	yes	Yes	yes
Macaulay [25]	yes	unclear	yes	no	no	yes	N/a	yes	no	yes	unclear	no	No	no
Regan[26]	yes	unclear	yes	yes	no	yes	N/a	yes	no	yes	unclear	unclear	Yes	yes
Reinhold [27]	yes	yes	unclear	no	no	yes	N/a	yes	no	Yes	unclear	yes	Yes	yes
Soto 1996[28]	no	unclear	unclear	yes	no	yes	N/a	yes	no	Yes	unclear	no	No	unclear
Soto 2000b[29]	yes	yes	unclear	no	no	yes	N/a	yes	unclear	Yes	yes	no	Yes	yes
Soto 2000a[30]	yes	yes	unclear	no	no	yes	N/a	yes	unclear	Yes	yes	no	Yes	yes
Stiris[31]	yes	unclear	unclear	yes	yes	yes	N/a	yes	no	yes	yes	unclear	Unclear	unclear
Sugiyama[32]	unclear	no	unclear	no	no	yes	N/a	yes	no	no	yes	yes	Yes	yes
Taylor[33]	yes	yes	unclear	yes	no	yes	N/a	yes	no	Yes	yes	unclear	Yes	yes
Textor[34]	yes	unclear	unclear	no	no	yes	N/a	yes	yes	Yes	unclear	no	Yes	yes
Varghese [35]	unclear	unclear	unclear	no	no	yes	N/a	yes	yes	yes	yes	yes	Yes	yes
Zidi[36]	yes	no	unclear	yes	no	yes	N/a	yes	yes	Yes	unclear	no	No	no

RS = reference standard (ERCP); IT = index test (MRCP).

QUADAS Questions

1. Was the spectrum of patients representative of the patients who will receive the test in practice?

2. Were selection criteria clearly described?

3. Is the reference standard likely to correctly classify the target condition?

4. Is the time period between reference standard and index test short enough to be reasonably sure that the target condition did not change between the two tests?

5. Did the whole sample or a random selection of the sample, receive verification using a reference standard of diagnosis?

6. Did patients receive the same reference standard regardless of the index test result?

7. Was the reference standard independent of the index test (i.e. the index test did not form part of the reference standard?

8. Was the execution of the index test described in sufficient detail to permit replication of the test?

9. Was the execution of the reference standard described in sufficient detail to permit its replication?

10. Were the index test results interpreted without knowledge of the results of the reference standard?

11. Were the reference standard results interpreted without knowledge of the results of the index test?

12. Were the same clinical data available when test results were interpreted as would be available when the test is used in practice?

13. Were uninterpretable/intermediate test results reported?

14. Were withdrawals from the study explained?

**Table 2:** The 25 included studies

**Study**	**Total number Of patients**	**Suspected Condition**	**True +ve**	**False +ve**	**False -ve**	**True -ve**	**N**	**Sensitivity (95% CI)**	**Specificity (95% CI)**	**Likelihood ratio-positive (95% CI)**	Likelihood ratio-negative (95% CI)*
Adamek[9]	86	Abnormality	42	1	5	12	60	0.89 (0.77, 0.95)	0.92 (0.67, 0.99)	11.62 (1.76, 76.57)	0.12 (0.02, 0.76)
Adamek	81	Malignancy	22	0	5	33	60	0.81 (0.63, 0.92)	1.00 (0.90, 1.00)	54.64 (3.47, 861.18)	0.20 (0.01, 3.14)
Angulo[11]	73	Normal	19	2	3	46	70	0.86 (0.67, 0.95)	0.96 (0.86, 0.99)	20.73 (5.28, 81.31)	0.14 (0.04, 0.56)
Angulo	70	dilitation CBD	38	2	3	27	70	0.93 (0.81, 0.97)	0.93 (0.78, 0.98)	13.44 (3.52, 51.33)	0.08 (0.02, 0.30)
Angulo	72	Obstruction	37	3	0	39	79	1.00 (0.91, 1.00)	0.91 (0.76, 0.97)	11.00 (3.74, 32.36)	0.00
Angulo	70	Choledocholithiasis	5	1	5	59	70	0.50 (0.24, 0.76)	0.98 (0.91, 1.00)	30.00 (3.90, 230.73)	0.51 (0.07, 3.91)
Angulo	70	PSC	19	1	4	46	70	0.83 (0.63, 0.93)	0.98 (0.89, 1.00)	38.83 (5.53, 272.37)	0.18 (0.03, 1.25)
Barish[12]	29	Dilitation	13	0	2	6	21	0.87 (0.62, 0.96)	1.00 (0.61, 1.00)	11.81 (0.81, 172.17)	0.17 (0.01, 2.45)
Calvo[13]	61	Choledocholithiasis	29	2	3	10	44	0.91 (0.76, 0.97)	0.83 (0.55, 0.95)	5.44 (1.53, 19.36)	0.11 (0.03, 0.40)
Chan[14]	47	Choledocholithiasis	18	4	1	22	45	0.95 (0.75, 0.99)	0.85 (0.66, 0.94)	6.16 (2.48, 15.26)	0.06 (0.03, 0.15)
Demartines[15]	40	Choledocholithiasis	19	2	0	19	40	1.00 (0.83, 1.00)	0.90 (0.71, 0.97)	10.50 (2.81, 39.24)	0.00
Dwerryhouse[16]	40	Choledocholithiasis	7	2	1	28	38	0.88 (0.53, 0.98)	0.93 (0.79, 0.98)	13.13 (3.35, 51.36)	0.1 (0.03, 0.52)
Guibaud[18]	79	Obstruction	72	0	7	47	126	0.91 (0.83, 0.96)	1.00 (0.92, 1.00)	87.00 (5.52, 1372.23)	0.09 (0.01, 1.49)
Guibaud		Choledocholithiasis	26	2	6	92	126	0.81 (0.65, 0.91)	0.98 (0.93, 0.99)	38.19 (9.60, 151.97)	0.19 (0.05, 0.76)
Guibaud		Malignancy	12	2	2	110	126	0.86 (0.60, 0.96)	0.98 (0.94, 1.00)	48.00 (11.96, 192.72)	0.15 (0.04, 0.58)
Holzknecht[20]	61	Choledocholithiasis	12	3	2	46	63	0.86 (0.60, 0.96)	0.94 (0.83, 0.98)	14.00 (4.58, 42.78)	0.15 (0.05, 0.47)
Holzknecht		Dilatation	32	2	2	26	62	0.94 (0.81, 0.98)	0.93 (0.77, 0.98)	13.18 (3.46, 50.23)	0.06 (0.02, 0.24)
Holzknecht		Stenosis	31	3	5	22	61	0.86 (0.71, 0.94)	0.88 (0.70, 0.96)	7.18 (2.46, 20.91)	0.16 (0.05, 0.46)
Holzknecht		Overall	43	3	4	12	62	0.91 (0.80, 0.97)	0.80 (0.55, 0.93)	4.57 (1.66, 12.63)	0.11 (0.04, 0.29)
Laokpessi[21]	101	Choledocholithiasis	105	0	8	34	147	0.93 (0.87, 0.96)	1.00 (0.90, 1.00)	64.78 (4.13, 1015.86)	0.08 (0.00, 1.19)
Lee[22]	46	Malignancy	17	2	4	23	46	0.81 (0.60, 0.92)	0.92 (0.75, 0.98)	10.12 (2.64, 38.86)	0.21 (0.05, 0.79)
Lomanto[23]	136	Choledocholithiasis	22	0	2	38	62	0.92 (0.74, 0.98)	1.00 (0.91, 1.00)	70.20 (4.46, 1105.97)	0.10 (0.01, 1.60)
Lomas[24]	76	Choledocholithiasis	9	2	0	58	69	1.00 (0.70, 1.00)	0.97 (0.89, 0.99)	30.00 (7.68, 117.19)	0.00
Lomas		Stricture	19	1	0	49	69	1.00 (0.83, 1.00)	0.98 (0.90, 1.00)	50.00 (7.18, 348.04)	0.00
Macaulay[25]	29	Dilatation	18	1	0	10	29	1.00 (0.82, 1.00)	0.91 (0.62, 0.98)	11.00 (1.70, 71.28)	0.00
Regan[26]	23	Choledocholithiasis	14	1	1	7	23	0.93 (0.70, 0.99)	0.88 (0.53, 0.98)	7.47 (1.19, 46.94)	0.08 (0.01, 0.48)
Reinhold[27]	110	Obstruction	69	0	7	34	110	0.91 (0.82, 0.95)	1.00 (0.90, 1.00)	63.18 (4.03, 991.27)	0.10 (0.01, 1.55)
Reinhold		Choledocholithiasis	27	3	3	77	110	0.90 (0.74, 0.97)	0.96 (0.90, 0.99)	24.00 (7.86, 73.31)	0.10 (0.03, 0.32)
Soto 1996[28]	46	Dilatation	26	1	1	16	44	0.96 (0.82, 0.99)	0.94 (0.73, 0.99)	16.37 (2.44, 109.77)	0.04 (0.01, 0.26)
Soto 2000[29]	57	Choledocholithiasis	23	1	1	24	49	0.96 (0.80, 0.99)	0.96 (0.80, 0.99)	23.96 (3.50, 163.78)	0.04 (0.01, 0.30)
Soto 2000[30]	51	Choledocholithiasis	25	0	1	25	51	0.96 (0.81, 0.99)	1.00 (0.87, 1.00)	49.11 (3.15, 765.62)	0.06 (0.00, 0.88)
Stiris[31]	50	Choledocholithiasis	28	1	4	17	50	0.88 (0.72, 0.95)	0.94 (0.74, 0.99)	15.75 (2.33, 106.28)	0.13 (0.02, 0.89)
Sugiyama[32]	159	anamalous PBJ	9	0	2	148	159	0.82 (0.52, 0.95)	1.00 (0.97, 1.00)	235.92 (14.60, 3811.82)	0.21 (0.01, 3.38)
Taylor[33]	146	Choledocholithiasis	45	9	1	74	129	0.98 (0.89, 1.00)	0.89 (0.81, 0.94)	9.02 (4.86, 16.74)	0.02 (0.01, 0.05)
Taylor		Stricture	12	1	0	118	131	1.00 (0.76, 1.00)	0.99 (0.95, 1.00)	118.00 (16.76, 830.78)	0.00
Textor[34]	150	PSC	29	1	4	108	142	0.88 (0.73, 0.95)	0.99 (0.95, 1.00)	95.79 (13.56, 676.70)	0.12 (0.02, 0.86)
Varghese[35]	191	Choledocholithiasis	31	3	3	154	191	0.91 (0.77, 0.97)	0.98 (0.95, 0.99)	47.72 (15.48, 147.06)	0.09 (0.03, 0.28)
Zidi[36]	70	Choledocholithiasis	28	0	21	21	70	0.57 (0.43, 0.70)	1.00 (0.85, 1.00)	25.08 (1.60, 392.61)	0.44 (0.03, 6.89)

* With likelihood ratios of zero, the data are unsuitable for the calculation of 95% confidence intervals.

**Table 3:** Study characteristics

**Study, country**	**Sample selection**	**Comparison (type of MRCP and reference tests used)**	**Description of patients and procedures; study period**	**Time between ERCP and MRCP**
Adamek et al, 1998; Germany[9]	Not reported	RARE and HASTE MRCP were compared with ERCP	86 patients entered the study; 8 were excluded due to biliary-enteric anastomoses, of the remaining 78, 16 had unsatisfactory ERCP, 2 had unsatisfactory MRCP (claustrophobia) leaving 60 patients who had both January-December 1996	Not reported
Angulo et al, 2000; USA[11]	Not reported	Fast spin echo pulse sequence compared with ERCP and PTC	Initially 74, 1 did not receive MRCP due to claustrophobia, 73 had MRCP, 68 had ERCP, 2 had PTC and 3 had neither Study period not stated	MRCP performed within 24 hours preceding the scheduled ERCP
Barish et al, 1995; USA[12]	Random selection from referrals	3D TSE MRCP compared with ERCP and PTC	30 patients initially selected, one patient did not receive MRCP due to the presence of ascitic fluid in the upper abdomen; three had PTC due to failed ERCP, 8 of the 29 patients did not have ERCP or PTC Study period not reported	ERCP was performed 8 hours after MRCP
Calvo et al, 2002; Spain[13]	Not reported	Two HASTE sequences MRCP compared with ERCP	116 patients with suspected biliopancreatic pathology initially, of these 61 patients were selected with suspected choledocholithiasis, failure in one patient for ERCP November 1996-February 1998	MRCP within 72 hours before ERCP
Chan et al, 1996; Hong Kong[14]	Consecutive sample	T2-weighted turbo spin-echo sequence (non-breath-hold, fat-suppressed) MRCP compared with ERCP	47 had MRCP, 45 had ERCP (two failures) May-August 1995	ERCP within 5 hours after MRCP
Demartines et al, 2000; Switzerland[15]	Not reported	3 acquisition techniques of MRCP were used including T2/T1 weighted, single-shot turbo spin echo and half-Fourier acquisition single-shot turbo spin echo heavy sequence compared with ERCP (high-risk patients) or intraoperative cholangiography (moderate risk patients)	40 patients received ERCP and MRCP and 30 received IOC and MRCP April 1997-September 1998	Not reported
Dwerryhouse et al, 1998; UK[16]	Not reported	T2 weighted TSE with non-breath-holding MRCP compared with ERCP and POC	Initially 405 patients who underwent laparoscopic cholecystectomy, of these 278 had no known risk factors for CB stones, 87 underwent early ERCP and were excluded. 40 patients with risk factors for CBD stones underwent MRCP. 2 patients had failed MRCP due to claustrophobia, ERCP was unsuccessful in 4 patients who then had peroperative cholangiography. February 1996 – January 1998	All patients underwent ERCP within 1 week after ERCP
Guibaud et al, 1995; Canada[18]	Consecutive	2D FSE MRCP compared with ERCP, PTC, T-tube cholangiography, surgery and autopsy	198 patients initially of which 72 were excluded due to no proof of bile duct obstruction (n = 42), unsuccessful ERCP (n = 12), unsuccessful MRCP due to claustrophobia (n = 6), inadequate ERCP (n = 10) or MRCP (n = 2) leaving 126 patients September 1992-March 1993	Time between MRCP and *final diagnosis *was less than 6 hours in 105 cases, less than 1 week in 15 cases and more than 1 week in six cases
Holzknecht et al, 1998; Germany[20]	Consecutive sample	RARE and half-Fourier RARE MRCP compared with ERCP	66 patients were eligible, 2 were excluded because of pacemakers, 3 had failed ERCP after MRCP leaving 61 patients who had both MRCP and ERCP June 1995 to April 1996	MRCP performed before ERCP (patients were due to have ERCP within the next 2 days)
Laokpessi et al, 2001; France[21]	Consecutive sample	FSE and T2 heavily weighted single-shot FSE sequences with fat suppression MRCP compared with ERCP or intraoperative cholangiography (IOC)	Initially 166 inpatients but only 147 patients had MRCP, of these 101 had ERCP and 45 had IOC and cholecystectomy. Those in group receiving ERCP had a past history of cholecystectomy or had a high surgical or anaesthetic risk. 21 removed from study for: refusal to sign protocol (n = 3), refusal to undergo MRCP (n = 4) or ERCP (n = 7), excessive time between MRCP and final diagnosis (n = 7) November 1997-December 1999	Average time between MRCP and final diagnosis 10 hours (range 3–48 hours) if longer than 48 hours between MRCP and final diagnosis, patients were removed from the study
Lee et al, 1997; South Korea[22]	Consecutive sample	3D steady-state free-precession MRCP compared with ERCP	71 patients of which 25 were excluded (8 because ERCP was not performed, 15 who were evaluated for intrahepatic stones, 1 for peripheral type of intrahepatic cholangiocarcinoma, 1 suspected mucinous ductal ectasia of the pancreas) leaving 46 patients who had both MRCP and ERCP January-March 1995	33 patients had MRCP before ERCP ranging from 6 hours to 5 days. The remaining 31 patients had ERCP first ranging from 3 to 16 days
Lomanto et al, 1997; Italy[23]	Not reported	T2 weighted TSE sequence MRCP compared with ERCP and PTC	136 patients referred for MRCP, of these 62 had MRCP for choledocholithiasis, (the other 74 were: 48 for stenosis of the biliary tract, 15 with previous hepaticojejunostomy and choledochojejunostomy and 11 with chronic pancreatitis) 60 of these patients had ERCP and 2 had PTC September 1994-October 1995	Not reported
Lomas et al, 1999; UK[24]	Not reported	Hybrid four-shot RARE (FSE) sequence and a single-shot half Fourier RARE sequence compared with ERCP	76 referrals, of these 2 did not have MRCP (one was obese and one was claustrophobic), 5 did not have ERCP (1 died, 1 refused and in 3 patients the operator was unable to cannulate the common bile duct) leaving 69 referrals in 66 patients 18 month period, dates not stated	MRCP took place first within 4 hours of ERCP
Macaulay, et al, 1995; USA[25]	Sequential	T2-weighted TSE MRCP (non-breath hold) compared with ERCP, PTC and IOC	28 patients initially had MRCP, 24 patients had 28 direct cholangiographic studies (21 had ERCP, 6 had PTC and 1 had IOC) Study period not reported.	ERCP took place within 1–4 hours in 15 patients, 4 were within 5–7 days after MRCP and 1 was 11 days before and another 109 days before MRCP, all PTC studies were within 2 days after MRCP and the 1 IOC preceded MRCP by 5 days.
Regan et al, 1996; USA[26]	Not reported	HASTE MRCP compared with ERCP and sonography	26 patients, 2 had unsuccessful ERCP and one did not have MRCP due to claustrophobia leaving 23 patients	MRCP was performed just before ERCP in 18 patients and within 24 hours in 5 patients
Reinhold et al, 1998; Canada[27]	Consecutive	FSE MRCP compared with ERCP, IOC and surgery	Initially 159 patients of which 49 were excluded due to the following reasons: 34 due to lack of diagnosis, 10 due to unsuccessful ERCP, 3 due to unsuccessful MRCP due to claustrophobia, inadequate ERCP (n = 1) or MRCP (n = 1) leaving a sample of 110 patients. 101 patients had ERCP, 2 had IOC and 7 had surgery. 5 month study period, dates not reported	MRCP was performed first and ERP or equivalent was less than 6 hours later in 97 patients, less than 1 week in 7 patients and more than 1 week in 6 patients
Soto et al, 1996; USA[28]	Randomly recruited	3D FSE MRCP compared with ERCP and PTC	46 patients, 7 of whom were included in Barish et al, 1995^58^, 45 had ERCP and 1 had PTC May 1994-April 1995	ERCP/PTC within 24 hours after MRCP
Soto et al, 2000a; Columbia[30]	Not reported	Breath hold, single shot half-Fourier rapid acquisition and non-breath-hold 3D FSE MRCP compared with ERCP, CT and oral-contrast enhanced CT cholangiography	Initially 68 patients, 12 did not meet inclusion or exclusion criteria, 2 did not have MRCP because of claustrophobia, in 3 ERCP was not attempted or completed leaving 51 patients who had all 4 studies. April 1998-March 1999	MRCP performed within 48 hours before ERCP
Soto et al, 2000b; Columbia[29]	Not reported	3D fast SE, Single section Half-Fourier RARE and multi-section half-Fourier RARE MRCP compared with ERCP	Initially 59 patients, 10 were excluded due to the following reasons: 2 due to MRCP contraindications, 4 because 1 or more of the 3 MRCP sequences could not be completed, and 4 because ERCP could not be completed August 1997-May 1998	MRCP was completed before ERCP within 72 hours
Stiris et al, 2000; Norway[31]	Consecutive sample	HASTE fat suppressed breath-hold MRCP compared with ERCP	50; all patients had both techniques; study period not stated	MRCP performed first followed by ERCP within 12 hours
Sugiyama et al, 1998; Japan[32]	Non consecutive	HASTE MRCP compared with ERCP	187 patients were recruited, 19 underwent only cholangiography or pancreatography on ERCP, in 8 the common channel could not be identified clearly and there was failure of cannulation in 2 patients leaving 159 patients with common bile duct, main pancreatic duct and common channel depicted June 1994-August 1996	MRCP was 0 to 14 days before ERCP
Taylor et al, 2002; Australia[33]	Consecutive sample	HASTE MRCP compared with ERCP, PTC or surgery	Initially 149 procedures (146 patients), MRCP unsuccessful in 8 due to claustrophobia and in 1 patient due to poor image quality, 5 were excluded because MRCP was more than 24 hours before ERCP, in 20 ERCP was unsuccessful (3 had subsequent ERCP, 2 had surgery and 2 had PTC and were included). In two patients ERCP and MRCP were both unsuccessful, leaving 129 patients who had both MRCP and ERCP (or equivalent). November 1998-December 1999	MRCP was performed within 24 hours before ERCP
Textor et al, 2002; Germany[34]	Consecutive sample	3D T2 weighted FSE MRCP compared with ERCP	150 patients initially, of which 146 had successful MRCP, 3 patients with PSC had unsuccessful ERCP and another failed due to a bilidigestive anastomosis January 1996-December 2000	ERCP was performed 1–14 days before MRCP (mean 3.2 days)
Varghese et al, 2000; Ireland[35]	Consecutive sample	T2 weighted 2D multi-slice FSE MRCP compared with ERCP, PTC or IOC	256 patients initially, 64 of which were excluded because ultrasound report or ERCP hard-copy images were not available (n = 30), direct cholangiography was not performed after failed ERCP (n = 22), MRCP not performed due to contraindications (n = 5), MRCP images were of non-diagnostic quality (n = 7), resulting in 191 patients [of these 34 had choledocolithiasis diagnosed by ERCP (n = 29), IOC (n = 3) and PTC (n = 2)] 18 month period, dates not stated	MRCP was performed before ERCP within a period of 4 hours to 2 weeks (mean 18 hours)
Zidi et al, 1999; France[36]	Consecutive sample	Non breath-hold fat suppressed TSE MRCP compared with ERCP (with or without sphincterotomy), endosonography or IOC	70 inpatients were included, 63 had ERCP, 5 had sonography and 2 had IOC 12 month period, dates not reported	MRCP performed within 12 hours before ERCP

**Table 4:** Studies comparing MRCP and ERCP with final diagnosis

**Study**	**Definition of Final diagnosis**	**Sensitivity**		**Specificity**	
		
		**MRCP vs. final diagnosis**	ERCP vs. final diagnosis*	**MRCP vs. final diagnosis**	ERCP vs. final diagnosis*
Adamek[9]	ERCP plus histological findings or follow-up	42/47 for any abnormality 0.89 (CI: 0.77–0.95) 22/27 for malignancy 0.81 (CI: 0.63–0.92)	0.91 for any abnormality 0.93 for malignancy	12/13 0.92 (CI: 0.67–0.99) for any abnormality 33/33 1.00 (CI: 0.90–1.00) for malignancy	0.92 for any abnormality 0.94 for malignancy
Laokpessi[21]	Stone extraction with ERCP or IOC	105/113 0.93 (CI: 87–0.96) for Choledocholithiasis	78/81 0.95 (CI: 0.87–0.98)	34/34 1.00 (CI: 0.90–1.00)	19/19 1.00 (CI: 0.79–1.00)
Lee[22]	ERCP plus surgical findings	17/21 for malignancy 0.81 (CI: 0.60–0.92)	15/21 0.71 (CI: 0.50–0.86)	23/25 0.92 (CI: 0.75–0.98)	23/24 0.96 (CI: 0.80 to 0.99)
Regan[26]	ERCP, endoscopic balloon or basket extraction or surgical removal of stones	14/15 0.93 (CI: 0.70–0.99) for choledocholithiasis	15/15 1.00 (CI: 0.80–1.00)	7/8 0.88 (CI: 0.53–0.98)	8/8 1.00 (CI: 0.68–1.00)

*Raw data was not reported in some studies, therefore it is not clear whether are not the two groups (MRCP vs. final diagnosis and ERCP vs. final diagnosis) are directly comparable.

**Table 5:** Estimates of diagnostic odds ratio (DOR), sensitivity and specificity from the regression of D = a + bS for Littenberg-Moses Summary ROC curve for MRCP test diagnosis of choledocholithiasis (n = 15 studies) for a range of values of diagnostic threshold (S)

	**Diagnostic Odds Ratio (DOR)**	**Sensitivity**	**Specificity**
mean S	193.22	0.91	0.95
min S	144.79	0.61	0.99
max S	239.34	0.97	0.87
